# Unravelling Metagenomics Approach for Microbial Biofuel Production

**DOI:** 10.3390/genes13111942

**Published:** 2022-10-25

**Authors:** Km Sartaj, Alok Patel, Leonidas Matsakas, Ramasare Prasad

**Affiliations:** 1 Department of Biosciences and Bioengineering, Indian Institute of Technology Roorkee, Roorkee 247667, Uttarakhand, India; 2 Biochemical Process Engineering, Division of Chemical Engineering, Department of Civil, Environmental, and Natural Resources Engineering, Luleå University of Technology, SE-971 87 Luleå, Sweden

**Keywords:** biofuels, metagenomics, enzyme, biomass, microbes

## Abstract

Renewable biofuels, such as biodiesel, bioethanol, and biobutanol, serve as long-term solutions to fossil fuel depletion. A sustainable approach feedstock for their production is plant biomass, which is degraded to sugars with the aid of microbes-derived enzymes, followed by microbial conversion of those sugars to biofuels. Considering their global demand, additional efforts have been made for their large-scale production, which is ultimately leading breakthrough research in biomass energy. Metagenomics is a powerful tool allowing for functional gene analysis and new enzyme discovery. Thus, the present article summarizes the revolutionary advances of metagenomics in the biofuel industry and enlightens the importance of unexplored habitats for novel gene or enzyme mining. Moreover, it also accentuates metagenomics potentials to explore uncultivable microbiomes as well as enzymes associated with them.

## 1. Introduction

To address the inadequate global energy supply and climate change concerns, much effort has been made towards minimizing the world’s dependency on conventional fossil fuel reserves as well as the generation of advanced technologies for sustainable fuel production [1]. Consequently, microbial-derived biofuels, such as biodiesel, ethanol, and butanol, have emerged as a promising solution and have attained growing interest from researchers, industry and policymakers due to their eco-friendly nature [2]. Owing to the capability to diminish these menacing concerns to a large extent, the interest in the aforementioned biofuel production using microbes has been steadily increasing in recent years. For example, U.S. and EU members both committed to using more renewable energy over conventional fuels to achieve net zero emissions by 2050 [3]. Even with the recent growth in global biofuel production, considerable technological bottlenecks still exist in the production processes to efficiently convert biomass into biofuels. The metabolic diversity of microorganisms enables them to produce biofuel via fermenting various substrates obtained from food crops (sugars, starch, canola seeds), lignocellulosic biomass from agricultural waste (sugar cane bagasse, corn stalks, switchgrass, poplar) and nutrient rich waste materials, etc. However, the food crops have sparked the “food versus fuel debate”, and, as such, non-food or feed plant biomass and waste materials should be used as feedstock for the microbial production of biofuels. Lignocellulosic biomass and waste material often require a pre-treatment step to break complex sugar molecules into fermentable ones or to minimize the toxicants in waste materials [4]. Nowadays, several thermochemical pre-treatment technologies are being successfully applied for this purpose, though implementation of environmentally hazardous organic solvents and high processing energy led to a sharp decline in their use. Thus, along with these technologies, industries decidedly consider enzymes a vital tool for biomass utilization and biofuel development because of their efficiency and selectivity in reaction chemistry [5]. Enzyme production has scaled up correspondingly; however, it has been found incompetent at meeting the rising demand for sustainable biofuels due to the unavailability of more robust strains, a complex production process, and the lack of infrastructure and expertise to manufacture beyond laboratory scale. To date, about 99.9% of microbes could not be discovered or isolated from the natural environment—a major limitation of the classical method of enzyme production (involve bacterial isolates culturing). Thus, tremendous research is being progressed worldwide to exploit unexplored microbial communities for identification and potent enzymes isolation [6].

Metagenomics is an advanced methodology that bypasses the requirement of the cultivation and isolation procedures allied with traditional microbial methods, and drastically broadens the spectrum of microbial resource utilization. It has become one of the powerful research tools for genetic engineering, environmental science, microbiology and biotechnology [7]. Countless reviews are available on every aspect of metagenomics, including sequencing strategies, functional metagenomics and biotechnological potential; nevertheless, they confer more towards varied frameworks for metagenomics-assisted isolation methods. Thus, the present article emphasizes metagenomics’ contribution as an emerging molecular technique for sustainable biofuel production (bioethanol and biodiesel both) concerning future technical and strategic improvements on a single platform, alongside deciphering advances in microbial metabolic engineering with the aid of metagenomics. Further, the article discusses the biotechnological implications as well as the metagenomic impact in shaping the socioeconomic landscape of the modern world.

## 2. Microbial Biofuel—An Overview

Fuels derived from biological materials, most often from plants or plant-based resources, with the use of microbes (bacteria, cyanobacteria, microalgae, yeast, fungi) and their enzymes are generally known as microbial biofuels. With the ability to combat against global climate change by reducing CO_2_ and other greenhouse gas emissions, microbial biofuels are being actively pursued as a potential renewable substitute for petroleum diesel. Thus, to enhance the security of national fuel supplies, the European Union, Brazil, and the United States have been implemented in a number of biofuel programs [8]. Depending on the feedstock sources, biofuels are classified as either first, second, third or fourth generation. Conventional or first-generation biofuel mainly includes the processing of food crops grown on arable land including starch, sugar, vegetable oil etc. Bioethanol is the most popular example of this generation that is produced from sugars (derived via enzymatic digestion of corn starch) or sucrose (obtained from sugarcane or sugar beets) fermentation and is widely applied in diverse sectors such as pharma, solvent industries, transportation etc. However, its economic incompetency and competition for land and water used for food production (food vs. fuel) are often listed as primary disadvantages [9]. To overcome these drawbacks, researchers turned their focus towards lignocellulosic biomass (grown on land not appropriate for food crops), waste agricultural residues, and organic fractions of municipal or household solid waste that give rise to second generation biofuel, which provides additional benefits for waste management improvement as well as fuel security. Algae-derived biofuels such as biodiesel are classified as the third generation while electro and photobiological solar fuels come under the category of fourth generation biofuels [10]. These two technologies are under development and not yet ready to be marketed at the industrial scale. 

In Europe, biodiesel is the most common biofuel generated by the transesterification of algal, vegetable oil and animal fats. Chemically, it encompasses long chain fatty acid methyl/alkyl esters and is used as a fuel for vehicles in its pure form (B100). Irrespective of standard diesel, biodiesel being oxygenated contains high oxygen and hydrogen content that leads to lower particulates and carbon monoxide emissions. However, utilization in its pure form may increase emissions of nitrous oxide, a greenhouse gas [11]. In terms of gaseous biofuels, biogas is a potential renewable energy source that can be upgraded to meet natural gas standards. Its production is a cyclic and continuous process including anaerobic digestion by methanogens. Besides zero carbon dioxide emissions, residual digestate (solid by product) generated during the process can be used as fertilizers. Biogas largely comprises carbon dioxide (CO_2_) and methane (CH_4_) which releases energy by combusting or oxidizing with oxygen thus biogas produced by advanced waste treatment technologies comprises 55–75% of methane [10]. Biobutanol is another promising biofuel, cited as a potential replacement for gasoline due to its direct utilization in internal combustion engines. It is mainly produced by an anaerobic fermentation process carried out by several microbial strains belonging to the genus *Clostridium* that yields acetone: butanol: ethanol in the ratio of 1:6:1. Moreover, unlike ethanol biobutanol, it shows high fuel density and less corrosivity, can be mixed in a high blend ratio called BUT16 and compatibility with existing petroleum distribution systems, including fuel pumps. Industrial investment in biobutanol is becoming more profitable as a genetically modified metabolic pathway enabled *Clostridium* to ferment agricultural, forest residues and suggests the feasibility of the second generation biobutanol industry [10,12]. 

Furthermore, amongst the above-discussed sustainable fuels, the focus towards microbes-derived second generation bioethanol and biodiesel (mostly obtained from lignocellulosic biomass and waste agricultural residues) production has been gradually increasing in the present-day. Thus, in line to advance their production process, new approaches are being developed continually. Genetic and metabolic engineering in microbes, multi-omics (transcriptomics, proteomics and metabolomics) tactics, nanotechnology-based biocatalyst synthesis (assist in transesterification reaction of biodiesel production), synthetic and system biology-based (includes metagenomics) approaches are few examples [13]. Metagenomics, used to study the structural and functional aspects of the entire nucleotide sequence isolated from varied environmental samples, is a new and thriving area and is likely to be an important contributor to resolving problems with renewable energy. Therefore, the next section discusses metagenomics and their foremost benefits for mainstream biofuel production. 

## 3. Introduction of Metagenomics

Metagenomics is a cutting-edge technology that emerged in the late 1990s and usually involves complete microbial genomic DNA extraction from specific environmental habitats, constructing libraries, and screening to seek novel functional genes or biologically active compounds [2,5]. This methodology plays a significant role in processing microbial genetic information that has not been cultivated before or that is difficult to culture in a laboratory. Similarly, available data can be utilized for new species discovery and to uncover earlier-identified biochemical pathways and functions. Thus, metagenomics offers a powerful tool for tapping the genetic and metabolic diversity of complex ecosystems [14,15]. 

**Figure 1:**
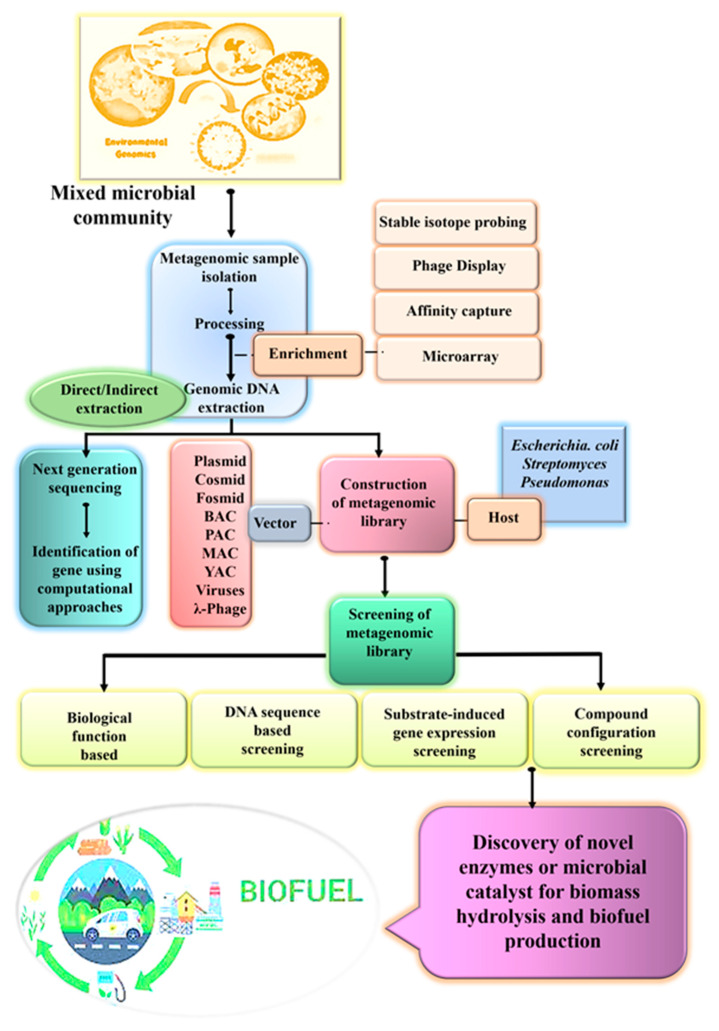
Overview of metagenomic approach for biodiesel production (image of biofuel is acquired from https://es.dreamstime.com/ciclo-de-vida-del-combustible-biol%C3%B3gico-image (accessed on 10 September 2022) 112,357,052).

Metagenomic studies typically follow a multistep strategy including the pre-treatment of samples containing environmental microorganisms, DNA extraction, proper host and vector selection for gene cloning as well as protein expression and further metagenomic library construction (functional metagenomic approach), sequencing, post metagenome sequencing (sequence-driven metagenomic approach), and data analysis. Amongst all steps, the isolation of pure, undegraded DNA from environmental samples is considered to be the most important and highly critical step. In view of this, numerous physical, chemical, mechanical and enzymatic methods for DNA material extraction are available. Those methods are categorized into the direct and indirect extraction methods. Direct methods include onsite cell lysis followed by the extraction and purification of metagenomic DNA, whereas microbial cells’ separation prior to lysis is vital for indirect methods. Direct methods are generally used for high DNA yield whereas the indirect method results in the high purity of DNA. Additionally, the overall metagenomic process relies on the development of molecular biology, microbiology and bioinformatics [16]. Through facilitating detailed learning of the microbial genome, metagenomics provides a platform for new gene discoveries—thus novel enzyme isolation that can be further screened for their different functional aspects like potential in antimicrobial agents, biochemically active and therapeutic compounds etc. (Figure 1).

## 4. Metagenomics—An Advanced Tool in Biofuel Sector

Recently in the biofuel sector, metagenomics is significantly contributing by discovering novel cell wall degrading enzymes that are key factors for lignocellulosic biomass depolymerization for subsequent conversion into biofuel and hence providing a cost-effective way to biofuel technology (Table 1).

Lignocellulosic biomass is mainly composed of cellulose, hemicellulose (fermentable polysaccharides) and lignin and can be hydrolyzed by various glycohydrolases on an industrial scale [2]. The recalcitrant nature of lignocellulosic materials and the constant need to further improve the efficiency of the hydrolysis enzymes necessitate microbial bioprospecting on extremophilic sites to identify unique cellulolytic microbes possessing high efficiency in biomass hydrolysis, required for biofuels commercialization. In this direction, Dimitra Zarafeta et al. (2016) introduced a robust GH5 cellulase termed CelDZ1 whose catalytic profile (activity at low pH, good thermostability, high halotolerance, resistance for metal ions and other denaturing agents) affirms its relevance towards ‘enzyme cocktails,’ mainly applied for the second-step processing of biomass in the biofuel industry. Briefly, the polysaccharide degrading microbe, *Thermoanaerobacterium,* was isolated via samples collected from Icelandic hot springs through an enrichment approach (anaerobic condition at pH 7, 55 °C with carbon source xylan and 0.01% yeast extract). Whole genomic DNA was extracted from these samples, sequenced via NGS (next-generation sequencing) and consecutively subjected to bioinformatic analysis in order to find sequences encoding for presumed cellulolytic enzymes. After ensuring enzyme sequence, amplified *celDZ1α* (via PCR, polymerase chain reaction) constructed into a plasmid pET-CelDZ1α that was cloned and overexpressed in *Escherichia coli* BL21(DE3) cells. Protein was purified, visualized by SDS-PAGE and Western blotting. Zymography, an electrophoretic technique for hydrolytic enzyme detection, applied further for qualitative analysis of cellulosic activity in which 0.25% CMC (carboxymethylcellulose) enriched 12% SDS-PAGE gel, was used. Enzyme activity was quantified by measuring the reducing sugar amount released from substrate (CMC) using the 3,5-dinitrosalicylic acid (DNS) method. The enzyme was further screened for optimal pH, temperature, substrate and salt concentrations and revealed structural features through X-ray crystallography provide potential explanations behind its biochemical characteristics [32]. New extremophilic cellulolytic eubacterium (*Caldicelluloseruptor bescii*) was also isolated from hot springs in the Valley of Geysers by implementing a metagenomic approach. A detailed study on its cell-free extracellular cellulase (CEC) represented its efficiency for the degradation of crystalline cellulose, various polysaccharides like xylan, non-pretreated plant biomass, and mannose-based substrates at high temperatures (≥75 °C–85 °C). Unlike *Trichoderma reesei* CEC (commonly used commercial cellulase) the degradation activities of *C. bescii* CEC for rice straw, timothy grass, and Avicel (microcrystalline cellulose) were found to be more effective, which suggests its usefulness in the processes operated at high temperatures > 75 °C [33,34]. Intending to identify novel bio-catalytic enzymes encompassing great efficiency to degrade cellulosic biomass gut microbiota are likely serving as a main target. For instance, bioinformaticians in NIAS (National Institute of Advanced Studies) explored goat rumen liquid and identified unique bifunctional cellulolytic gene sequences. From the hindgut of wood-feeding higher termite (*Nasutitermes corniger*), 886 proteins were identified among which 197 show known enzymatic functions. To perform this metagenomic study, collected termites from the Costa Rica were first morphologically classified on the basis of cytochrome oxidase gene sequence and dissected in an anterior P3 hindgut compartment within 36 h of collection. Luminal contents (about 165 specimens) were pooled, diluted with buffered saline and frozen immediately. Further, DNA extraction was performed and inserted containing whole genome shotgun libraries prepared as well as sequenced with BigDye Terminator v3.1. From these libraries, fosmid vectors were prepared which were completely sequenced using traditional and pyrosequencing methods. All collected data were incorporated into the Integrated Microbial Genomes with Microbiome Samples (IMG/M) system. Aliquots encompassing centrifuged luminal content and protein from soluble fraction were heat-treated, reduced, alkylated, digested and analyzed by LC-MS/MS and were searched against the termite metagenome database with SEQUEST (program for mass spectrometry data analysis). Identified glycosyl hydrolases (GHs) were named as CAZy (carbohydrate-active enzymes) and were thoroughly screened for their activity with microcrystalline and phosphoric-acid-swollen cellulose (PASC) [33,35]. Moreover, functional screening of metagenomic libraries from distinct microbiomes also directed the purification and characterization of several cellulolytic enzymes from three distinct cellulolytic sites; extreme environmental samples, rabbit’s cecum and lake water microbiomes [33]. Additionally, a novel endoglucanase showing stability on a broad range of pH (3.5–10.5), enzymes such as β-glucosidase (Bgl1A, Bgl1D) were also reported from marine and soil metagenomes, respectively. Bgl1A finds significant industrial application owing to its high glucose tolerance and low sensitivity to product inhibition, and Bgl1D was found to be highly active at low temperatures, pH (5.5–10.5) and high ionic liquid titer. In the case of Bgl1A, microbial samples were preferably collected from South China Sea’s surface water. The BAC (Bacterial Artificial Chromosome) library using vector pIndigoBAC-5 was prepared with extracted DNA and transformed into the *E. coli* EPI300. After screening of positive clones (forms black halo around colonies on LB agar plate supplemented esuculin hydrate, ammonium ferric, chloramphenicol), plasmid accommodating β-glucosidase gene retransformed into *E. coli* EPI300 by electroporation. From these positive clones, BAC DNA was extracted and recombinant plasmid vector pUC19 constructed and transformed into *E. coli* DH5α with the next steps being isolation and the sequencing of inserted fragments. Further, the β-glucosidase gene *bgl1A* was PCR-amplified and subjected to overexpression using recombinant plasmid pET22b-bgl1A encompassing *E. coli BL21*(DE3) as the host. Protein purified and enzyme activity were tested by pNPG (p-Nitrophenyl β-D glucopyranoside) as a substrate. Likewise, for Bgl1D, DNA was extracted from alkaline-polluted soil in Southern China and a metagenomic library was constructed by the pGEM-3Zf (+) cloning vector with *E. coli* DH5α as a host. Further, DNA was sequenced and sequences allied with the gene *bgl1D* were identified and overexpressed in *E. coli* Tuner (DE3)pLacI by forming a recombinant plasmid pGEXC106. Lineweaver–Burk plot (Enzyme kinetic program) was used to determine enzymes kinetic parameters Vmax and Km [36,37,38]. Several carbohydrate-modifying enzymes (three endoglucanase, one β-cellobiohydrolase, 2β-glucosidase) were mined in metagenomic libraries from casts and guts of earthworms (*Lumbricus terrestris* and *Aporrectodea caliginosa*) collected from Moscow State University, Russia. Casts of each species were obtained on wet sterile filter paper through which suspension was prepared with sterile distilled water. The aqueous phase of this suspension was used to inoculate the Getchinson medium (details provided by Beloqui et al. (2010)). Only the liquid phase was considered for DNA extraction. A fosmid library was prepared with pCCFOS vector and *E. coli* EPI300-T1 as host. GHs sequences were identified, amplified by PCR and cloned into the pET-30-Ek/LIC vector and then introduced into *E. coli* BL21 (DE3)pLysS for expression [39].

A library created from insect gut microbiomes through the NGS approach as later screened for xylanase activity. In addition to the successful cloning of cold-active xylanase (Xyn8), highly active, substrate specific endo-acting alkaline xylanase (Xyl6E) has also been retrieved from a library of microbiome extracted from fungus-growing termites [40,41,42]. Moreover, lignin degradation has been achieved by several peroxidases; lignin, manganese and phenol-oxidases, isolated by the metagenomic-derived approach [43,44].

Metagenomic libraries prepared from intertidal flat sediments of coastal regions, South Korea revealed the presence of lipase—LipEH166—a major contributor in biodiesel production and also employed for lipid-rich wastewater bioremediation obtained from several industries such as dairy, cosmetics, food, detergents etc. Additionally, to search cold-active lipases, cold-sea sediment samples were also screened. Subsequently, the fosmid clone was selected and the whole sequence was determined by shotgun sequencing. Data analysis reveals the presence of 25 open reading frames (ORF) among which ORF20 (EML1) was found to be similar to lipases. The gene for EML1 was expressed in *E. coli* and extracted protein purified by metal-chelating chromatography. A temperature of 25 °C and a pH of 8.0 were found to be optimal for enzymatic activities. Unique cold-adapted alkaline lipase, LipCE, also comes in this series [45,46,47]. Acidic (EstPS2, lipolytic activity at 37 °C, pH 5) and alkaline (Est2K, lipolytic activity at 50 °C, pH 10.0) esterase were recovered from peat swamp forest soil and a compost metagenomic library, respectively [46,48]. Various thermostable (Est1−70 °C; EstE1−80 °C), as well as cold-adapted (EstM-N2; EstM-N1) esterase, have been derived from thermal environments and arctic soil samples, respectively. In detail, samples from the Jae Sawn hot spring at 70 °C temperature and pH 7 were taken for thermostable esterase Est1 isolation. Post-extraction and purification, the DNA was fractioned (1–10 kb) with *Sau*3AI, ligated to pZErO-2 vector and transformed into *E. coli* TOP10 by electroporation. Transformed colonies were selected and screened for esterase activity (on LB agar plate encompassing 1% trioleoylglycerol and Rhodamine B dye). Bacterial colonies displaying positive results on screening were selected for plasmid DNA isolation and sequencing. A comparative study was performed with multiple sequences of bacterial lipases and esterase (retrieved from protein and nucleotide databases on NCBI Entrez server). BLASTX was performed for sequence similarity searches, the phylogenetic analysis was performed with the Align X program. Further, the enzyme was expressed by amplicon cloning into pET-28a(+) and insertion in *E.coli* BL21(DE3)(pLysS). Protein was extracted from selected transformants and Hi-Trap affinity column containing nickle-nitrilo-triacetic acid used for purification and subjected to SDS-PAGE analysis. Enzymatic activity was determined by the spectrophotometric method and includes p-nitrophenol quantification released after lipolytic-catalyzed hydrolysis. By following a similar approach, EstE1 enzyme isolated from the hot springs of different regions of Indonesia and a library was constructed by pCC1FOS fosmid vector that was packaged in lambda packaging extracts and transformed into an EPI300-T1R phage T1-resistant *E. coli* host. After screening and DNA sequencing, PCR-amplified amplicon ligated with expression vector pET-22b(+) and introduced into *E. coli* BL21(DE3). Transformants were cultivated followed by extraction and protein purification. For cold-adapted esterase, the metagenomic library constructed with arctic region soil samples using fosmid vectors (CopyControl^TM^ or pEpiFOS^TM^-5 fosmid library production kit). DNA from positive fosmid clones were isolated and ligated with pUC19, transformed into *E. coli* DH5α and examined for lipolytic activity. Standard PCR reaction was performed after that product was cloned into the pCR2.1-TOPO vector and sequence to ensure correct gene amplification. Restriction digested DNA fragments ligated into the pQE30 and constructed expression plasmids pQE30-EstM-N1 and pQE30-EstM-N2 were transformed into *E. coli* JM109 cells by electroporation. Histidine tagged EstM-N1 and EstM-N2 enzymes purified by immobilized metal affinity chromatography and esterase activity measured as described above [49,50,51]. Ferrer et al. (2005) stated different clones possessing cyclodextrinase activity that was further utilized for cyclodextrin hydrolysis into maltose or glucose [44]. The novel esterolytic enzyme (EstDZ2) isolated by the screening of a metagenomic sample collected from a hot spring in Kamchatka, Russia, displayed high activity against medium chain fatty acid esters at 25–60 °C and pH 7–8. Exquisite stability against a high concentration of organic solvents was also a characteristic feature of this enzyme [32].

The discovery of many other useful enzymes in biofuel production such as amylases, alcohol oxidoreductases, alcohol/aldehyde dehydrogenase, has also been led by metagenomics [52,53]. Novel cold-tolerant endoglucanase (CelCM3, significant activity at 4 °C), isolated by the metagenomic analysis of camel rumen, can serve as a bioremediating agent by resisting metal ions, detergents and other organic solvents. These aforementioned enzymes are highly valuable in downstream applications, hence they revolutionized second-generation biofuel production by cutting down the costs of pre-treatment processes and thus helping to commercialize the technology.

## 5. Metagenomics and Enzyme Engineering Assisted Upscaling of Biofuel Production

Metabolic engineering strategies including carbon flux modulation towards target pathways, co-factor engineering for an adequate supply of NADH/NADPH, codon optimization, incorporating heterologous pathways, promotor engineering, competitive pathway deletion, consolidated pathway insertion, directed enzyme evolution, tailoring toxic metabolite tolerance and respiratory pathway manipulation, are currently being employed to refine microbial metabolic pathways towards advanced biofuel production [54]. Amongst several approaches, enzyme-targeted methods considered as the most crucial step in achieving the goal of high-titer and productivity. Thus, the current section provides information about how metagenomics along with enzyme engineering assist biofuel upscaling. For instance, native *Saccharomyces cerevisiae,* the most apposite host for bioethanol production, was not able to metabolize atypical substrates such as xylose (found up to 40% in lignocellulosic hydrolysate). However, to attain a high ethanol yield from lignocellulosic biomass necessitate co-fermentation of all sugar types present in hydrolysate. Among several attempts made in this direction, metagenomics-derived isolation of gene encoding Xylose isomerase (XI) enzyme is the one. Bingyin et al. (2015) extracted metagenomic DNA from soil, snail manure from Singaporean natural forests and XI genes from mammal gut microbes, *Alistipes* sp. HGB5, actively expressed in a respiration-deficient *S. cerevisiae* strain that results in maximal xylose conversion and high, specific ethanol productivity (0.283 g g_CDW_^−1^ h^−1^) [55]. Two other XI genes (*xym1, xym2*) were also isolated from the soil metagenomic library. However, their enzymatic activities expressed in *S. cerevisiae* were comparable to *Piromyces* XI (known for high XI activity) but the aerobic growth rate was found to be very low. In this regard, the activity of XI genes (Ru-*xyl*A) isolated from the bovine rumen metagenomics library was improved by mutagenesis (site-directed mutation in K11T/D220V) and functionally expressed in *S. cerevisiae* [56,57]. The substitution of asparagine residue at position 337 of novel XI, RsXI-C1 (from protist residing in hindgut of termite *Reticulitermes speratus*) with cysteine improved xylose utilization ability of yeast 2.5 times more than without mutation. As the recombinant strain allows much lower xylitol accumulation, this provides the additional benefits of cofactor imbalance that could impede metabolic flux [58]. A psychrohalophilic Xyn-2, novel endo-1,4-xylanase from the camel rumen metagenome, is a successful hydrolysis agricultural waste; wheat bran-100%, rice straw-56%, wheat straw 56%, rice bran 41%. Ethanologenic *Bacillus subtilis* AP is able to produce 5.5 g/L ethanol with 22.6% yield in consolidated bioprocessing (CBP) alongside this enzyme. Additionally, in the SSF system (simultaneous saccharification and fermentation), this may have a great impact with 7.3 g/L concentration and 26.8% ethanol yield [59]. The hydrolysis of autoclaved pre-treated rice straw (cellulose rich most abundant agricultural waste) in the presence of PersiCel4, endo-β-1,4-glucanase from the sheep rumen metagenome increased the reducing sugar content by up to 260% after 72 h in very harsh conditions (temperature 85 °C, pH 8.5) [60]. Another cellulase, Cel-5M from the rumen metagenome identified as the GH5 family endoglucanase, was tested for lignocellulosic biomass hydrolysis especially wheat straw. An ethanol yield of 0.46 g/g and 0.43 g/g cellulase was obtained after alkali- and steam-treated wheat straw respectively. This multifunctional enzyme also showed activity on various substrates such as CMC, Xylan, Avicel, Glucopyranoside, β-Mannan and filter paper [61]. Moreover, the heterologous expression of endoglucanase Cel776 (from hot spring of Spain) in *S. cerevisiae* enabled the strain to degrade cellulose and xylan both [62]. In a very new approach for cellulose hydrolysis, magnetic nanobiocatalyst was prepared by way of the covalent immobilization of cellulase CelDZ1 (described in Section 4), horseradish peroxidase (HRP), β-glucosidase (bgl) and glucose oxidase (GOx) on the surface of amino-functionalized magnetic nanoparticles. In a cascade of reaction, CelDZ1 hydrolyze cellulose into cellobiose converts into glucose via bgl. Further, glucose oxidation forms gluconic acid and H_2_O_2_ that act as a substrate for HRP, reduced to form H_2_O [63]. Metagenomically isolated cellulase from pig ordure and rice straw, Exo2b was applied to improve cellulase activity in *T. reesei* a cellulase-producing fungi. In brief, the fragment-encoding Exo2b catalytic domain fused with the *cbh1* gene from *T. reesei* and was successfully expressed in *T. reesei* Rut-C30. Compared to the parental strain, CMCase activity in secreted proteins was increased by 18% and high glucose concentration by 19.8% was attained by secreted protein-mediated hydrolysis of corn stover [64]. A crude extract of LipC12, lipase obtained by metagenomics immobilized on Immobead 150 (Ibead-CELipC12) and applied for the enzymatic synthesis of biodiesel as 85% oleic acid conversion was observed in 48 h [65]. In order to form immobilized lipase artificial body AOB-sole-lip-1, a new gene encoding lipase (*Lip-1*) extracted from metagenomic BAC library constructed via water samples from hot springs, China was cloned and fused with the oleosin gene, and overexpressed in *E. coli*. The monomeric Sole-lip-1 fusion protein showed transesterification activity at a 45 °C optimal temperature. Saturated and polyunsaturated methyl esters were obtained after enzymatic transesterification of olive oil and methanol [66].

## 6. Metagenomic Implications on Biotechnology

Microbial cultivation to produce limitless biological compounds, including antibiotics, enzymes, vitamins, amino acids, various biomaterials, low molecular weight substrates etc., is the most predominant area of industrial biotechnology. Thus, by evolving unculturable microbial species, metagenomics opens new horizons in the development of biotechnology as well as provides new opportunities for searching novel gene families and gene products [67]. As per the previous discussion, the enzymes produced from this technique showed valuable properties, such as stability at low or high temperatures, high activity in acidic or alkaline conditions, resistance to high saline conditions, heavy metals, organic solvents, pressure and radiation are much needed for the industrialization of any biotechnological process. Metagenomics studies working in the direction of pathway-centric approaches have also been applied to provide insight about the microbial community interaction in a specific environment. In this context, an interesting work linked to the functional gene exploration in leaf-cutter ant fungus gardens should be highlighted. By virtue of the metagenomic approach, enzymes and pathways involved in the symbiosis between leaf-cutter ants and their cultivar have been determined with the advent of novel cellulases [35].

## 7. Future Perspectives and Challenges

Even though metagenomics has been proven an efficient technology in microbial process development, bottlenecks still need to be resolved. For instance, advancement in heterologous gene expression originating from unculturable microorganisms is required, as the majority of enzymes are still uncharacterized. It is challenging to understand the exact role of an enzyme identified from complex environmental microbial communities, which mandates the strengthening of the combination of metagenomics and bioinformatics technologies. The development of high-throughput cloning methods is needed to efficiently express soluble protein, as well as further progression in some novel approaches such as metatranscriptomics, structural genomics must be initiated. Moreover, to access complex metagenomic sequence information, bioinformatic and mining programs such as “metagenome Mapserver, ANASTASIA” need to be established [68,69]. Yet, to assure sustained growth in the metagenomics field, more innovative and sophisticated bioinformatics tools must be developed. With continuous development in all these areas, metagenomics technology will be the most important tool for studying microbial diversity in the natural environment and the screening of new genes or biologically active substances.

## 8. Conclusions

In day-to-day life, the demand for renewable energy has been continuously increasing, hence breakthrough research is being conducted in this field. Metagenomics allow for the mining of cellulose-degrading enzymes that potentially break down lignocellulosic biomass into fermentable sugars, allowing the identification of more efficient enzymes that, in turn, facilitate the transition to economically feasible biofuel production. Furthermore, the newest development in metagenomics, including metatranscriptomics and metaproteomics, promises the advanced screening of uncultivated microbes and establishes metagenomics as a great tool to access inaccessible organisms. Apart from playing a crucial role in the bioenergy field, metagenomics can also be successfully applied in several other arenas such as in the bioremediation and health sector (known as clinical metagenomics that includes the genomic analysis of samples collected from patients), but their full potential in mentioned fields still remains to be unraveled.

## Figures and Tables

**Table 1:** Enzymes discovered through metagenomic approach along with isolation site/screening method and industrial applications.

Enzyme	Source	Special Feature/Screening Method	Application	References
Cellulase	Buffalo rumen	Thermotolerant	Bioethanol, biopolishing of textile fibers	[17]
Cellulase	Anaerobic beer	Halotolerant	Bioethanol, biodiesel, extraction of flavoring materials and essential oils	[18]
Hemicellulase	Degrading wheat straw	Thermotolerant, halotolerant	Bioethanol, biodiesel, bioplastic from wood biomass	[19]
Xylanase	Sea-bottom	Sequence based	Bioethanol	[20]
Xyloglucanase	Bovine rumen	Activity based	Bioethanol	[21]
Acidobacterial-laccase like copper oxidase	Marsh bog soil	Thermostable, halotolerant, stable in the presence of organic solvents	Polyphenol removal from wine, bioethanol production	[22]
Esterase	Antarctic soil	Function based (fosmid vector)	Bioethanol production	[23]
Glycoside hydrolases	Anaerobic digestion sludge	Sequence based	Bioethanol production	[24]
Cyclodextrinase	Bovine rumen	Function based	Biodiesel production	[25]
Enzymes involved in bioremediation				
Naphthalene dioxygenase	Oil-contaminated soil	Function based	Bioremediation of oil-contaminated soil/water	[26]
Oxygenases	Artificially polluted soil	Function based	Remediation of oil-contaminated soil/water	[27]
Laccase	Water from South China Sea	Sequencing based	Dye degradation	[28]
Phenol hydroxylases and catechol 2,3-dioxygenases	Wastewater treatment plant	Function based	Potential use in aromatic compound degradation	[29]
Enzymes used in other applications				
Lipase/protease/hemolysins/biosurfactants	Slaughterhouse drain	Function based	Applied as antimicrobial agent	[30]
β-Glucosidase	Hydrothermal spring water	Function based	Thermotolerant and heath-active enzyme	[31]

## Data Availability

Not applicable.

## References

[1] Patel A., Sarkar O., Rova U., Christakopoulos P., Matsakas L. (2021). Valorization of volatile fatty acids derived from low-cost organic waste for lipogenesis in oleaginous microorganisms-A review. Bioresour. Technol..

[2] Xing M., Zhang X., Huang H. (2012). Application of metagenomic techniques in mining enzymes from microbial communities for biofuel synthesis. Biotechnol. Adv..

[3] European Commission (2022). Joint Statement between the European Commission and the United States on European Energy Security.

[4] Patel A., Arora N., Sartaj K., Pruthi V., Pruthi P.A. (2016). Sustainable biodiesel production from oleaginous yeasts utilizing hydrolysates of various non-edible lignocellulosic biomasses. Renew. Sustain. Energy Rev..

[5] Madhavan A., Sindhu R. (2017). Metagenome Analysis: A Powerful Tool for Enzyme Bioprospecting. Appl. Biochem. Biotechnol..

[6] Liu S., Moon C.D., Zheng N., Huws S., Zhao S., Wang J. (2022). Opportunities and challenges of using metagenomic data to bring uncultured microbes into cultivation. Microbiome.

[7] Biddle J.F., Fitz-gibbon S., Schuster S.C., Brenchley J.E., House C.H. (2008). Metagenomic signatures of the Peru Margin subseafloor biosphere show a genetically distinct environment. Proc. Natl. Acad. Sci. USA.

[8] Hill J., Nelson E., Tilman D., Polasky S., Tiffany D. (2006). Environmental, economic, and energetic costs and benefits of biodiesel and ethanol biofuels. Proc. Natl. Acad. Sci. USA.

[9] Mohr A., Raman S. (2015). Lessons from first generation biofuels and implications for the sustainability appraisal of second generation biofuels. Energy Policy.

[10] Ramos J.L., Pakuts B., Godoy P., García-Franco A., Duque E. (2022). Addressing the energy crisis: Using microbes to make biofuels. Microb. Biotechnol..

[11] Subhash G.V., Mohan S.V. (2011). Biodiesel production from isolated oleaginous fungi *Aspergillus* sp. using corncob waste liquor as a substrate. Bioresour. Technol..

[12] Guo Y., Liu Y., Guan M., Tang H., Wang Z., Lin L., Pang H. (2022). Production of butanol from lignocellulosic biomass: Recent advances, challenges, and prospects. RSC Adv..

[13] Rai A.K., Al Makishah N.H., Wen Z., Gupta G., Pandit S., Prasad R. (2022). Recent Developments in Lignocellulosic Biofuels, a Renewable Source of Bioenergy. Fermentation.

[14] Datta S., Rajnish K.N., Samuel M.S., Pugazlendhi A., Selvarajan E. (2020). Metagenomic applications in microbial diversity, bioremediation, pollution monitoring, enzyme and drug discovery. Environ. Chem. Lett..

[15] Alves L.D.F., Westmann C.A., Lovate G.L., Marcelino G., De Siqueira V., Borelli T.C., Guazzaroni M. (2018). Metagenomic Approaches for Understanding New Concepts in Microbial Science. Int. J. Genom..

[16] Wang H., Hart D.J., An Y., Hart D.J. (2019). Functional Metagenomic Technologies for the Discovery of Novel Enzymes for Biomass Degradation and Biofuel Production. Bioenergy Res..

[17] Shah R.K., Patel A.K., Davla D.M., Parikh I.K. (2017). Molecular cloning, heterologous expression, and functional characterization of a cellulolytic enzyme (Cel PRII) from buffalo rumen metagenome. 3 Biotech.

[18] Yang C., Xia Y., Qu H., Li A.D., Liu R., Wang Y., Zhang T. (2016). Discovery of new cellulases from the metagenome by a metagenomics—Guided strategy. Biotechnol. Biofuels.

[19] Maruthamuthu M., Jiménez D.J., Stevens P., Van E.J.D. (2016). A multi-substrate approach for functional metagenomics-based screening for (hemi) cellulases in two wheat straw-degrading microbial consortia unveils novel thermoalkaliphilic enzymes. BMC Genom..

[20] Hung K.S., Liu S.M., Tzou W.S., Lin F.P., Pan C.L., Fang T.Y., Sun K.H., Tang S.J. (2011). Characterization of a novel GH10 thermostable, halophilic xylanase from the marine bacterium *Thermoanaerobacterium saccharolyticum* NTOU1. Process Biochem..

[21] Findley S.D., Mormile M.R., Sommer-Hurley A., Zhang X.C., Tipton P., Arnett K., Porter J.H., Kerley M., Stacey G. (2011). Activity-Based Metagenomic Screening and Biochemical Characterization of Bovine Ruminal Protozoan Glycoside Hydrolases. Appl. Environ. Microbiol..

[22] Ausec L., Berini F., Casciello C., Cretoiu M.S., van Elsas J.D., Marinelli F., Mandic-Mulec I. (2017). The first acidobacterial laccase-like multicopper oxidase revealed by metagenomics shows high salt and thermo-tolerance. Appl. Microbiol. Biotechnol..

[23] Fu C., Hu Y., Xie F., Guo H., Ashforth E.J., Polyak S.W., Zhu B., Zhang L. (2011). Molecular cloning and characterization of a new cold-active esterase from a deep-sea metagenomic library. Appl. Microbiol. Biotechnol..

[24] Xia Y., Ju F., Fang H.H.P., Zhang T. (2013). Mining of Novel Thermo-Stable Cellulolytic Genes from a Thermophilic Cellulose-Degrading Consortium by Metagenomics. PLoS ONE.

[25] Ferrer M., Martínez-Abarca F., Golyshin P.N. (2005). Mining genomes and “metagenomes” for novel catalysts. Curr. Opin. Biotechnol..

[26] Ono A., Miyazaki R., Sota M., Ohtsubo Y., Nagata Y., Tsuda M. (2007). Isolation and characterization of naphthalene-catabolic genes and plasmids from oil-contaminated soil by using two cultivation-independent approaches. Appl. Microbiol. Biotechnol..

[27] Nagayama H., Sugawara T., Endo R., Ono A., Kato H., Ohtsubo Y., Nagata Y., Tsuda M. (2015). Isolation of oxygenase genes for indigo-forming activity from an artificially polluted soil metagenome by functional screening using *Pseudomonas putida* strains as hosts. Appl. Microbiol. Biotechnol..

[28] Fang Z., Li T., Wang Q., Zhang X., Peng H., Fang W., Hong Y., Ge H., Xiao Y. (2011). A bacterial laccase from marine microbial metagenome exhibiting chloride tolerance and dye decolorization ability. Appl. Microbiol. Biotechnol..

[29] Silva C.C., Hayden H., Sawbridge T., Mele P., De Paula S.O., Silva L.C.F., Vidigal P.M.P., Vicentini R., Sousa M.P., Torres A.P.R. (2013). Identification of Genes and Pathways Related to Phenol Degradation in Metagenomic Libraries from Petroleum Refinery Wastewater. PLoS ONE.

[30] Thies S., Rausch S.C., Kovacic F., Schmidt-Thaler A., Wilhelm S., Rosenau F., Daniel R., Streit W., Pietruszka J., Jaeger K.E. (2016). Metagenomic discovery of novel enzymes and biosurfactants in a slaughterhouse biofilm microbial community. Sci. Rep..

[31] Schröder C., Elleuche S., Blank S., Antranikian G. (2014). Characterization of a heat-active archaeal β-glucosidase from a hydrothermal spring metagenome. Enzym. Microb. Technol..

[32] Zarafeta D., Kissas D., Sayer C., Gudbergsdottir S.R., Ladoukakis E., Isupov M.N., Chatziioannou A., Peng X., Littlechild J.A., Skretas G. (2016). Discovery and Characterization of a Thermostable and Highly Halotolerant GH5 Cellulase from an Icelandic Hot Spring Isolate. PLoS ONE.

[33] Bilal T., Malik B., Rehman K. (2018). Metagenomic analysis of uncultured microorganisms and their enzymatic attributes. J. Microbiol. Methods.

[34] Kanafusa-shinkai S., Wakayama J., Tsukamoto K., Hayashi N., Miyazaki Y., Ohmori H., Tajima K., Yokoyama H. (2013). Degradation of microcrystalline cellulose and non-pretreated plant biomass by a cell-free extracellular cellulase/hemicellulase system from the extreme thermophilic bacterium *Caldicellulosiruptor bescii*. J. Biosci. Bioeng..

[35] Aylward F.O., Burnum K.E., Scott J.J., Suen G., Tringe S.G., Adams S.M., Barry K.W., Nicora C.D., Piehowski P.D., Purvine S.O. (2012). Metagenomic and metaproteomic insights into bacterial communities in leaf-cutter ant fungus gardens. ISME J..

[36] Duan C., Xian L., Zhao G., Feng Y., Pang H., Bai X., Tang J., Ma Q., Feng J. (2009). Isolation and partial characterization of novel genes encoding acidic cellulases from metagenomes of buffalo rumens. J. Appl. Microbiol..

[37] Fang W., Liu J., Hong Y., Peng H., Zhang X., Sun B., Xiao Y. (2010). Cloning and characterization of a β-glucosidase from marine microbial metagenome with excellent glucose tolerance. J. Microbiol. Biotechnol..

[38] Jiang C., Li S., Luo F., Jin K., Wang Q., Hao Z., Wu L., Zhao G., Ma G., Shen P. (2011). Biochemical characterization of two novel β-glucosidase genes by metagenome expression cloning. Bioresour. Technol..

[39] Beloqui A., Nechitaylo T.Y., Lo N., Polaina J., Strittmatter A.W., Reva O., Waliczek A., Yakimov M.M., Golyshina O.V., Ferrer M. (2010). Diversity of Glycosyl Hydrolases from Cellulose-Depleting Communities Enriched from Casts of Two Earthworm Species. Appl. Environ. Microbiol..

[40] Brennan Y., Callen W.N., Christoffersen L., Dupree P., Goubet F., Healey S., Herna M., Keller M., Li K., Palackal N. (2004). Unusual Microbial Xylanases from Insect Guts. Appl. Environ. Microbiol..

[41] Lee C.C., Kibblewhite-accinelli R.E., Wagschal K., Robertson G.H., Wong D.W.S. (2006). Cloning and characterization of a cold-active xylanase enzyme from an environmental DNA library. Extreamophiles.

[42] Liu N., Yan X., Zhang M., Xie L., Wang Q., Huang Y., Zhou X., Wang S., Zhou Z. (2011). Microbiome of fungus-growing termites: A new reservoir for lignocellulase genes. Appl. Environ. Microbiol..

[43] Ye M., Li G., Liang W.Q., Liu Y.H. (2010). Molecular cloning and characterization of a novel metagenome-derived multicopper oxidase with alkaline laccase activity and highly soluble expression. Appl. Microbiol. Biotechnol..

[44] Beloqui A., Pita M., Polaina J., Martı A., Golyshina O.V., Zuma M., Yakimov M.M., Garcı H., Alcalde M., Ferna M. (2006). Novel polyphenol oxidase mined from a metagenome expression library of bovine rumen biochemical properties, structural analysis, and phylogenetic relationship. J. Biol. Chem..

[45] Kim E., Oh K., Lee M., Kang C., Oh T., Yoon J. (2009). Novel cold-adapted alkaline lipase from an intertidal flat metagenome and proposal for a new family of bacterial lipases. Appl. Environ. Microbiol..

[46] Jeon J.H., Kim J., Kim Y.J., Kim H., Lee H.S., Kang S.G., Kim S., Lee J. (2009). Cloning and characterization of a new cold-active lipase from a deep-sea sediment metagenome. Appl. Microbiol. Biotechnol..

[47] Elend C., Schmeisser C., Hoebenreich H., Steele H.L., Streit W.R. (2007). Isolation and characterization of a metagenome-derived and cold-active lipase with high stereospecificity for (R)-ibuprofen esters. J. Biotechnol..

[48] Bunterngsook B., Kanokratana P., Tanapongpipat S., Uengwetwanit T., Rachdawong S., Vichitsoonthonkul T., Anapongpipat S.T., Engwetwanit T.U., Achdawong S.R. (2010). Identification and characterization of lipolytic enzymes from a peat-swamp forest soil metagenome. Biosci. Biotechnol. Biochem..

[49] Rhee J.K., Ahn D.G., Kim Y.G., Oh J.W. (2005). New thermophilic and thermostable esterase with sequence similarity to the hormone-sensitive lipase family, cloned from a metagenomic library. Appl. Environ. Microbiol..

[50] Tirawongsaroj P., Sriprang R., Harnpicharnchai P., Thongaram T., Champreda V., Tanapongpipat S. (2008). Novel thermophilic and thermostable lipolytic enzymes from a thailand hot spring metagenomic library. J. Biotechnol..

[51] Yu E.Y., Kwon M., Lee M. (2011). Isolation and characterization of cold-active family VIII esterases from an arctic soil metagenome. Appl. Microbiol. Biotechnol..

[52] Wexler M., Bond P.L., Richardson D.J., Johnston A.W.B. (2005). A wide host-range metagenomic library from a waste water treatment plant yields a novel alcohol/aldehyde dehydrogenase. Environ. Microbiol..

[53] Knietsch A., Waschkowitz T., Bowien S., Henne A., Daniel R. (2003). Construction and Screening of Metagenomic Libraries Derived from Enrichment Cultures: Generation of a Gene Bank for Genes Conferring Alcohol Oxidoreductase Activity on *Escherichia coli*. Appl. Environ. Microbiol..

[54] Joshi S., Mishra S.D. (2022). Recent advances in biofuel production through metabolic engineering. Bioresour. Technol..

[55] Peng B., Huang S., Liu T., Geng A. (2015). Bacterial xylose isomerases from the mammal gut Bacteroidetes cluster function in *Saccharomyces cerevisiae* for effective xylose fermentation. Microb. Cell Fact..

[56] Parachin N.S., Gorwa-Grauslund M.F. (2011). Isolation of xylose isomerases by sequence-and function-based screening from a soil metagenomic library. Biotechnol. Biofuels.

[57] Hou J., Shen Y., Jiao C., Ge R., Zhang X., Bao X. (2016). Characterization and evolution of xylose isomerase screened from the bovine rumen metagenome in *Saccharomyces cerevisiae*. J. Biosci. Bioeng..

[58] Katahira S., Muramoto N., Moriya S., Nagura R., Tada N., Yasutani N., Ohkuma M., Onishi T., Tokuhiro K. (2017). Screening and evolution of a novel protist xylose isomerase from the termite *Reticulitermes speratus* for efficient xylose fermentation in *Saccharomyces cerevisiae*. Biotechnol. Biofuels.

[59] Rajabi M., Nourisanami F., Ghadikolaei K.K., Changizian M., Noghabi K.A., Zahiri H.S. (2022). Metagenomic psychrohalophilic xylanase from camel rumen investigated for bioethanol production from wheat bran using *Bacillus subtilis* AP. Sci. Rep..

[60] Ariaeenejad S., Sheykh Abdollahzadeh Mamaghani A., Maleki M., Kavousi K., Foroozandeh Shahraki M., Hosseini Salekdeh G. (2020). A novel high performance in-silico screened metagenome-derived alkali-thermostable endo-β-1,4-glucanase for lignocellulosic biomass hydrolysis in the harsh conditions. BMC Biotechnol..

[61] Patel M., Patel H.M., Dave S. (2020). Determination of bioethanol production potential from lignocellulosic biomass using novel Cel-5m isolated from cow rumen metagenome. Int. J. Biol. Macromol..

[62] Escuder-Rodríguez J.J., González-Suarez M., deCastro M.E., Saavedra-Bouza A., Becerra M., González-Siso M.I. (2022). Characterization of a novel thermophilic metagenomic GH5 endoglucanase heterologously expressed in *Escherichia coli* and *Saccharomyces cerevisiae*. Biotechnol. Biofuels Bioprod..

[63] Giannakopoulou A., Patila M., Spyrou K., Chalmpes N., Zarafeta D., Skretas G., Gournis D., Stamatis H. (2019). Development of a Four-Enzyme Magnetic Nanobiocatalyst for Multi-Step Cascade Reactions. Catalysts.

[64] Geng A., Zou G., Yan X., Wang Q., Zhang J., Liu F., Zhu B., Zhou Z. (2012). Expression and characterization of a novel metagenome-derived cellulase Exo2b and its application to improve cellulase activity in *Trichoderma reesei*. Appl. Microbiol. Biotechnol..

[65] Madalozzo A.D., Martini V.P., Kuniyoshi K.K., De Souza E.M., Pedrosa F.O., Glogauer A., Zanin G.M., Mitchell D.A., Krieger N. (2015). Immobilization of LipC12, a new lipase obtained by metagenomics, and its application in the synthesis of biodiesel esters. J. Mol. Catal. B Enzym..

[66] Yan W., Li F., Wang L., Zhu Y., Dong Z., Bai L. (2017). Discovery and characterizaton of a novel lipase with transesterification activity from hot spring metagenomic library. Biotechnol. Rep..

[67] Shestakov S.V. (2012). Impact of Metagenomics on Biotechnological Development. Appl. Biochem. Microbiol..

[68] Li L., Mccorkle S.R., Monchy S., Taghavi S., Lelie D.V.D. (2009). Bioprospecting metagenomes: Glycosyl hydrolases for converting biomass. Biotechnol. Biofuels.

[69] Koutsandreas T., Ladoukakis E., Pilalis E., Zarafeta D., Kolisis F.N., Skretas G., Chatziioannou A.A. (2019). ANASTASIA: An Automated Metagenomic Analysis Pipeline for Novel Enzyme Discovery Exploiting next Generation Sequencing Data. Front. Genet..

